# Effect of Emotion, Expectation, and Privacy on Purchase Intention in WeChat Health Product Consumption: The Mediating Role of Trust

**DOI:** 10.3390/ijerph16203861

**Published:** 2019-10-12

**Authors:** Ming-Yan Wang, Peng-Zhu Zhang, Cheng-Yang Zhou, Neng-Ye Lai

**Affiliations:** 1 Department of Information Management, Management School, Shanghai University of Engineering Science, Shanghai 201620, China; wmycindy2020@126.com (M.-Y.W.); 13916826525@163.com (C.-Y.Z.); 15157107949@126.com (N.-Y.L.); 2 Department of Management Information System, AnTai College of Economics & Management, Shanghai Jiao Tong University, Shanghai 200030, China

**Keywords:** healthy consumption, purchase intention, trust, emotional support, expectation confirmation, privacy concern

## Abstract

With the aging of the population and the upgrading of the consumption structure of national health demand in China, it has become a new trend for the public to actively seek health products and services on social networks. Based on the theory of reasoned behavior and the theory of expectancy confirmation, this study aims to analyze the cognitive factors and their effects on WeChat users’ purchase intention in the process of health product consumption. Considering that safety is a key feature of health products that distinguishes them from other consumer products, the “satisfaction” concept in the expectancy confirmation model is replaced by “trust” in this study. Two hundred and two (202) valid samples were collected by a questionnaire survey to analyze their intentions to buy health products on WeChat. Theoretical models and corresponding research hypotheses were verified by structural equation modeling. The research results show that emotional price and emotional experience are positively correlated with trust and purchase intention. There is an obvious negative correlation between privacy invasion and trust. Expectation confirmation is positively associated with trust. Moreover, the intermediary test shows that trust has completely mediated between emotional price and purchase intention, and trust also has a full intermediary effect on expectation confirmation and purchase intention.

## 1. Introduction

With the growth of urbanization and industrialization in China, the impacts on residents’ lifestyle, ecological environment and food safety on health are gradually becoming apparent. Adolescent sub-health aging and chronic diseases have become urgent public health problems which could hinder the improvement of the health level of Chinese residents. Meanwhile, with the increase of disposable income of Chinese residents, the public’s health awareness is also increasing, and the consumption and demand for health services are also gradually improving. How to meet the needs of public healthcare management and promote the development of the health industry has become an inevitable requirement for the sustainable and healthy development of the economy and society. According to the implementation of the “Healthy China Strategy”, China’s health care industry has been further promoted. In the new situation, the “Chinese-style health industry system” has been restructured, and the health care industry is the whole healthy chain guided by the new objective of improving people’s health literacy, accepting scientific health guidance and reasonable health consumption. The health care industry involves many productions and service fields closely related to human health, and it has become an emerging industry with huge market opportunities. Consumption of health-related has become the mainstream consumption trend in China and the world. Conventional health products include medical products, health care products, nutrition products, healthcare management. A broader range of health products also includes functional food and nutritional food. The Chinese public is actively developing a healthy lifestyle and actively seeking health-related products and medical services through the Internet. With the development of mobile commerce, health products have been sold on the WeChat platform since 2014.

The combination of the Internet and the health care industry is an inevitable trend of today’s development. Online health consumption could increase consumers’ medical knowledge and more access to medical information, meanwhile, it also promotes health care quality [1,2]. Through network information technology, healthcare management services can be better provided for the public. Mobile commerce technology based on Web 2.0 plays a key role in the field of mobile health(m-health) and promotes the mobile socialization of healthcare management [3,4]. Digital media can provide users to find health information and potentially improve the chances of finding health content [5]. Users can obtain health information and purchase health products on the WeChat platform. According to the 39th report on China’s Internet Network Information Center (CNNIC), as of December 2016, the scale of internet medical users was 195 million, accounting for 26.6% of online users, with an annual growth rate of 28.0% in China. Online counseling and online purchasing of health products and services account for about 6 percent of internet users [6]. 

The application of Internet and mobile application technology in healthcare management will have a great impact on consumers’ health cognition and behavior. Consumers’ health needs will affect their purchase intentions, emotional responses, and preferences of health products. Health-conscious consumers are more concerned about their healthy diet, so they actively change their health behaviors, for example, by being more willing to buy organic food [7]. Nowadays, social networks play an important role in health care. The web-based social health system can provide opportunities to expand medical knowledge and increase participation in individual healthcare management [8]. Health behaviors based on the social network are mainly health information seeking and health-related social support [9]. Health behaviors on social networks are related to emotional support and health self-efficacy, emotional support is the most common dimension in the Facebook environment [10]. Social relationships established in cyberspace can readily provide the public with self-managed medical staff, disease information and emotional support [11,12]. Emotional support is an emotional response to the experience of using products and services [13]. Customers’ perception of service quality and service environment will have an impact on their emotional satisfaction, which will also change their beliefs and attitudes, and then affect their decisions [14,15]. Consumers’ trust tendency, personal privacy concerns, website information quality, and brand reputation may all affect consumers’ purchase intention of mobile commerce [16,17]. Health products such as nutritional supplement have a good reputation for corporate social responsibility, consumers will generate positive emotions and increase their willingness to buy healthy products [18,19].

The studies have clearly indicated that there is a relationship between the intention of buying health products and cognition of emotion. The process of changing consumers’ attitudes towards products or brands also reflects their emotional reactions [20]. It is found that adding emotional attributes to brand marketing can increase consumers’ participation and perceived differentiation, thus enhancing the stability of consumers’ choice. Emotions in shopping online process of consumers include happiness, awakening, and domination, these emotions will have an impact on consumers’ purchasing behavior before cognition [21]. If consumers can choose the product conveniently through the website functionality and have fun in the process of use and achieve the expected effect, these experience will stimulate their positive emotions, otherwise, it will cause negative effects [14,22]. Emotional design of products mainly starts from consumers’ experience level and emotional needs. Emotional design is an important aspect of new product development, and it is also important to improve customer satisfaction with new products [23]. In the context of online consumption, sensory information generated by emotional design will have a significant impact on consumers’ attitudes and preferences [24].

Trust seems to be a more critical factor of any high involvement in online consumption [25]. The online health community has become a valuable platform for patients to communicate and find support. However, health products of the network context cannot be tested online, so it would have a negative impact on trust in their purchasing decision [26]. Online consumers’ perceived trust or risk can have a significant impact on their purchasing decisions, moreover, online consumers’ perceived risks of products and web vendors have almost the same effect on trust [27]. Moreover, it is found that trust plays a fully mediating role in the relationship between perceived corporate reputation and purchase intention [28].

Many companies use information technology to process and mine consumer information to improve customer satisfaction, but sometimes these interventions can cause privacy concerns [29,30]. With the development of online healthcare management, social media could collect and store more information about users, the potential privacy threat brought by system insecurity is considered to be the invasion of users’ privacy [31,32]. In recent years, a variety of mobile health apps have been installed on smartphones for users to use. Previous studies have found that users worry about apps collecting personal health and activity information [33,34]. People rarely share health information with their friends, they don’t want health information to be accessed by third parties [35]. Moreover, privacy concerns can reduce trust and enhance risk awareness, thus indirectly affecting users’ willingness to use information system [35,36]. 

Users will have initial expectations on products and services before a purchase. If perceived value and satisfaction increase, consumers’ purchase intention will also increase [37]. Based on the expectation-confirmation theory, the positive expectation is positively correlated with satisfaction, and positive expectation will also increase the post-usage benefits [38]. Expectation confirmation could affect the willingness to use information system continuously through perceived availability and system satisfaction [39]. Perceived usefulness and perceived value will have an impact on mobile app stickiness and purchase intention, [40,41]. In the process of network business, if the perception of product experience or product usefulness is far lower than the expectation, then the satisfaction of consumers will be reduced [42,43]. 

WeChat has become the central hub of China’s mobile network. WeChat is a network of strong emotional relationships, which could establish various relationships, such as classmates, colleagues, and friends. WeChat has become an emerging business platform for Chinese medical and health industry and even overseas purchasing and marketing of health products. Due to the strong relationship between users in social networks, the emotional and cognitive involvement of users in social networks will raise the purchase intention of recommended products in the circle of friends [44]. This shows that how to consider the emotional needs of consumers, increase the sense of trust, and promote the consumption of health products is a new problem worth studying in the WeChat strong relationship circle. In the pages that follow. This study will analyze how the cognitive behavior of Chinese users using WeChat affects their intention of healthy consumption. In this paper, health products on WeChat Commerce system studied in this article refer to the broad definition of the term, such as traditional Chinese and Western medicines (over the counter medicines), health therapy, health care underwear and weight loss, nutritional products, functional foods and so on. The emotional support involved in this paper is mainly the emotional response generated by users when they use WeChat social media service. Considering that the safety of health products is more important than other products, the trust might better express consumers’ emotional cognition than satisfaction. Based on the Theory of Reasoned Action (TRA) and the Theory of Expectancy Confirmation (ECT), this paper establishes a cognition-trust-intention theoretical model. Data from the study were collected from WeChat users who had purchased health products. The theoretical model and corresponding research hypothesis are tested by structural equation modeling to explore the cognitive factors and influencing mechanism of users’ purchase intention. It is hoped that this research will make a contribution to a deeper insight into the purchasing behavior of health products in social networks through empirical and theoretical research. It could improve the competitiveness of pharmaceutical enterprises to some extent and bring new impetus to the promotion of public healthcare management.

## 2. Research Hypothesis and Theoretical Model

### 2.1. Emotional Support and Purchase Intention

Rational and emotional factors for products or services play an important role in purchasing decision [45]. Emotional support in a social commerce environment allows consumers to actively overcome difficulties and seek answers to their inner perceptions of sellers, products or services, therefore, consumers may have a desire to purchase [46]. If consumers form emotional cognition in the social and business environment, it may have an impact on consumers’ purchasing decisions [47]. Some studies found that the sensory design of products and brand experience will affect consumers’ perception and will trigger participation in purchasing in decision-making [48,49]. In the online process of pharmaceutical sales, the main reasons for users to buy medicines online are price and convenience [50]. Moreover, consumers’ pre-sale and after-sales service experience have a significant positive correlation with satisfaction and repurchase intention [51]. Emotional support can also influence the willingness to buy health products of WeChat. Based on the research reviewed, the study will attempt to verify this fact by the following hypotheses:

**Hypotheses 1 a** **(H 1a.)** *Emotion price is positively associated with purchase intention.*

**Hypotheses 1 b** **(H 1b.)** *Emotion product is positively associated with purchase intention.*

**Hypotheses 1 c** **(H 1c.)** *Emotion experience is positively associated with purchase intention.*

### 2.2. Emotional Support and Trust

Emotional support affects the formation of trust in social commerce [52,53]. Moreover, emotional support may significantly affect relationship satisfaction and trust [54]. The packaging design of products will affect consumers’ health perception and judgment of product function and value [55,56]. Consumers’ perception of the effectiveness and good experience in business systems will affect their attitudes and willingness to actively seek for disease information and accomplish healthcare management on the Internet [57,58]. Moreover, the response time, service content and interaction depth on the online medical platform will affect the trust and satisfaction of consumers on the online medical treatment [59]. Therefore, this study proposes the following hypotheses:

**Hypotheses 2 a** **(H 2a.)** *Emotion price is positively associated with trust.*

**Hypotheses 2 b** **(H 2b.)** *Emotion product is positively associated with trust.*

**Hypotheses 2 c** **(H 2c.)** *Emotion experience is positively associated with trust.*

### 2.3. Privacy Concern and Trust

Privacy is an important factor for users to accept using the information system for healthcare management services. Consumers regard disclosure of privacy without consent will decrease trust and make them no longer anonymous [60,61]. In particular, medical information is considered to be very sensitive personal information. Once private information is disclosed, it will threaten the data integrity and security of users, which may lead to malicious attacks on users [62]. Network users have certain expectations for network privacy, especially for the protection of information from unknown third parties [63,64]. In social networks, disclosure of private information will affect the trust of users, therefore, people will develop an attitude of resistance when they perceive that their privacy and freedom are controlled by others [65,66].

Consumer acceptance of mobile shopping applications can be affected by location sensitivity and risk [67]. Scholars have not conducted in-depth studies on the privacy factors of the WeChat. But “LBS+” opened a new model of WeChat marketing. The function of “find peoples nearby” may also be used by businesses to advertise for free, which may generate the disclosure of personal information at the same time. Based on previous research and the characteristics of privacy concern between the WeChat platform, this study defines that the privacy concern in the marketing process of WeChat health products is mainly affected by three factors: (1) Perceived monitoring refers to personal concerns that the WeChat system may monitor private information such as location or mobile phone; (2) Perceived intrusion refers to the concern of users that their privacy is received by too many business applications or illegally acquired by third-party platforms in the business process; (3) Information disclosure refers to user’s concern that their privacy may be used for other business purposes without permission. Thus, the following three hypotheses were developed:

**Hypotheses 3 a** **(H 3a.)** *Perceived monitoring is negatively associated with trust.*

**Hypotheses 3 b** **(H 3b.)** *Perceived intrusion is negatively associated with trust.*

**Hypotheses 3 c** **(H 3c.)** *Perceived information disclosure is negatively associated with trust.*

### 2.4. Expectation Confirmation and Trust

Some studies show that when a trusted party demonstrates a level of reliability consistent with consumer expectations, consumers’ trust will increase, moreover, consumers’ willingness to buy products is mainly driven by perceived value, trust, and satisfaction [68,69]. Consumer trust is a prerequisite for establishing an online market for healthy products such as green products and nutrition, which have an important influence on purchasing decisions [70,71]. Consumers’ perceived trust and expectation confirmation have an important impact on consumers’ purchase intention, and a significant positive correlation is found between consumers’ trust and expectation [72,73]. Based on the above discussions, hypothesis 4 is stated as:

**Hypotheses 4** **(H 4.)** *Expectation confirmation is positively associated with trust.*

### 2.5. Trust and Purchase Intention

Based on the theory of organizational trust, the establishment of organizational trust in the context of e-commerce will affect purchase intention [74]. Organizational trust is meaningful to the establishment of economic relations between unfamiliar parties, which will maintain a significant supportive impact on consumers’ purchase intention [75]. Trust in social networking sites (SNS) would increase the need for information seeking, which in turn increases the familiarity and social presence of the platform [76]. When consumers decide to provide private information on social media platforms, their trust mainly depends on the credibility of merchants [77]. Accordingly, this paper proposes that:

**Hypotheses 5** **(H 5.)** *Trust in the process of consumers’ purchase intention is positively associated with consumers’ purchase intention.*

### 2.6. The Mediating Role of Trust Between Emotional Support and Purchase Intention

Network trust in e-commerce has a significant relationship with perceived privacy, perceived service quality and repurchase intention [78]. The enterprises could strengthen users’ trust in their brands through emotional input, which had a positive impact on users’ purchase intention [79]. In order to meet a higher level of consumer demand, emotional experience and product packaging play an increasingly effect on purchasing decisions [24]. At the same time, website content and website sex will have an impact on network user trust, and trust in the effectiveness of network marketing shows an intermediary function [80]. Product information, quality, and price in product attributes have a supportive effect on purchase intention [81]. So, the paper will verify the facts with the following hypotheses:

**Hypotheses 6 a** **(H 6a.)** *An intermediary effect of trust is shown between emotion price and its purchase intention.*

**Hypotheses 6 b** **(H 6b.)** *An intermediary effect of trust is shown between emotion product and its purchase intention.*

**Hypotheses 6 c** **(H 6c.)** *An intermediary effect of trust is shown between emotion experience and its purchase intention.*

### 2.7. Expectation Confirmation and Purchase Intention

Expectation confirmation plays important roles in purchasing behavior. Expectation confirmation and perceived value will significantly affect consumers’ purchasing decisions [82]. When consumers buy a product for the first time, their purchase decision is based on the expectation created by the brand and packaging design of the product, or the previous experience of the relevant product [83,84]. By actively assuming social responsibilities, enterprises will improve consumers’ expectation identification of enterprises and enhance their willingness to buy relevant products [85]. Accordingly, we propose that:

**Hypotheses 7** **(H 7.)** *Expectation confirmation is positively corrected with consumers’ purchase intention.*

### 2.8. The Mediating Role of Trust Between Expectation Confirmation and Purchase Intention

To improve online transactions, online retailers need to focus on measures to build and maintain consumer trust. The role of expectation shows a positive correlation with consumer trust [86,87]. In the electronic market, consumers make purchasing decisions based on trust and expectation of products or enterprises [70,88]. The quality of products and purchasing experience of online retailers will gain confidence in products and generate a sense of trust [89]. 

Referring to the preceding research, the following hypothesis is proposed for this study:

**Hypotheses 8** **(H 8.)** *An intermediary effect of trust is shown between expectation confirmation and purchase intention.*

### 2.9. Proposed Model

**Figure 1:**
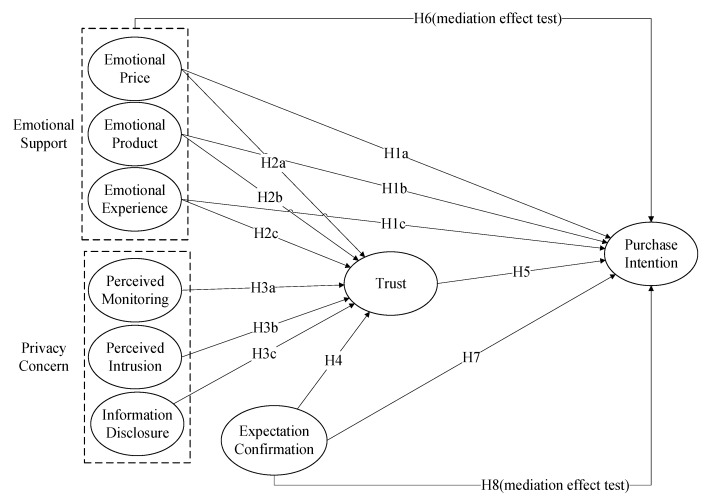
Theoretical model and hypotheses.

The model of this study is proposed by referring to the theory of reasoned action and the theory of expectancy confirmation, which combines with previous studies and the definition of the above hypotheses. In this study, emotional support, privacy concern, expectation confirmation are taken as an independent variable, consumer trust is taken as an intermediary variable, and consumers’ purchase intention is chosen as a dependent variable (Figure 1).

## 3. Method

### 3.1. Questionnaire Design and Analysis Method

This paper uses a 5-point Likert scale. In the design of the questionnaire survey. The verification of each variable should be measured using a minimum of three items to ensure the rationality of the questions [90]. Moreover, when factor analysis and structural equation model are carried out, the measurement items corresponding to each variable are less than 3, which may lead to unsatisfactory structural validity. The specific measurement questions about the questionnaire variables are summarized in Table 1. In the following research, variable names are abbreviated for ease of expression and calculation as shown in Table 1.

Structural equation modeling can estimate the relationships between multiple and interrelated variables. This method has the ability to deal with unobservable assumptions in the model, such as trust, expectation and other variables that cannot be directly measured. It can also analyze the structural relationships between potential factors. In this study, the method was used to examine the relationship between emotional support (ES), privacy concern (PC), expectation confirmation (EC), trust (T), and purchase intention (PI).

### 3.2. Profiling of the Sample 

The main purpose of this study is to analyze the influence of individual purchase behaviors of health products on purchase intention in social networks. This study analyzes consumers with WeChat mobile network-shopping experience. A total of 212 questionnaires were collected in the form of paper and online questionnaires. There were 202 valid questionnaires, and the effective rate of questionnaires was 95.3%. A total of 89 male samples (44.1%) was collected from the survey and female samples were 113 (55.9%). From the age of the interviewed groups, users aged 20–31 accounted for 76.7% of the total number of surveyed users, and social media services have a higher penetration rate among the young user groups. In terms of education level, 96.5% of the respondents have a bachelor degree or above. The age distribution, education level and economic status of the population in this study can significantly reduce the number of variables introduced in the model, which is conducive to the establishment of a simplified analysis model.

## 4. Results 

### 4.1. Reliability and Validity Test of the Questionnaire 

According to the results of Table 2, the square root of AVE of the latent variables is higher than the correlation coefficient between various factors, which proves that the questionnaire about WeChat users’ intention to buy health products has a good discriminant [96].

Cronbach alpha coefficient was used to test the reliability of the questionnaire. According to the calculation, the Cronbach alpha coefficients of all variables were greater than 0.7, indicating that the reliability of the questionnaire was good (see the results in Table 3). 

In terms of validity, we conducted an exploratory factor analysis on the questionnaire and found that KMO= 0.897 and sig=0.000, which is suitable for factor analysis. We used AMOS25 for confirmatory factor analysis and found that the factor loads of all variables were greater than 0.5, CR>0.7, and AVE>0.5 indicating good validity of the questionnaire (see the results in Table 3). 

### 4.2. Model Fit

Table 4 shows that the indexes of the model meet the requirements, and the fitting is reasonable.

### 4.3. Second-Order Factor Analysis

**Figure 2:**
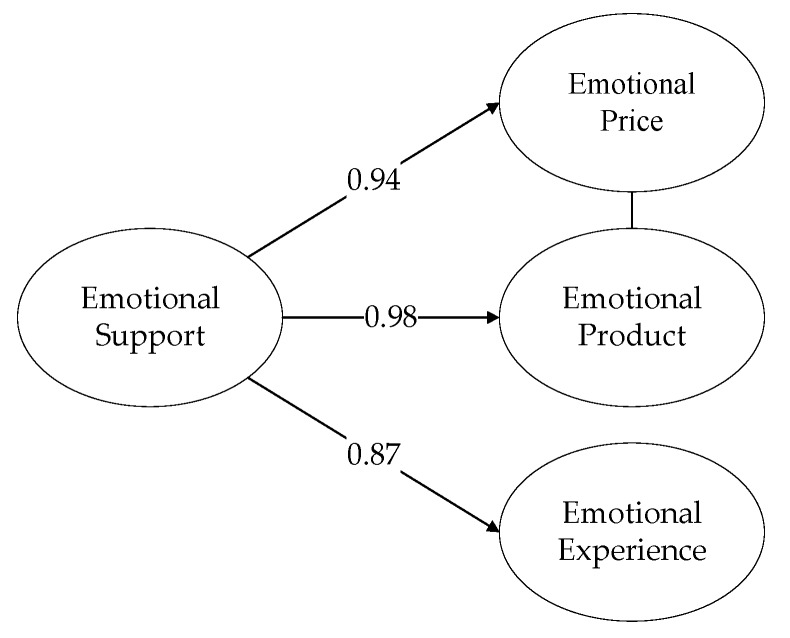
Emotion Support (ES).

**Figure 3:**
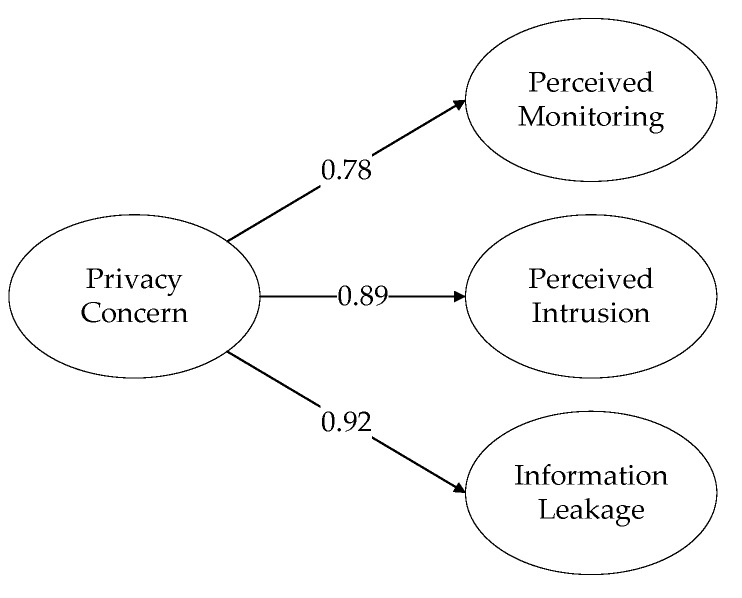
Privacy Concern (PC).

The concept of Emotion Support (ES) in the social network services is constructed from three dimensions: Emotional Price (EP1), Emotional Product (EP2), and Emotional Experience (EE). The concept of Privacy Concern (PC) in the social network services is constructed from three dimensions: Perceived Monitoring (PM), Perceived Intrusion (PI1), and Information Disclosure (ID). Figure 2 and Figure 3 show that all the factor load greater than 0.7, which means the constructions of Emotion Support (ES) and Privacy Concern (PC) are reasonable.

### 4.4. Structural Model Relationships Analysis

According to the Table 5, we found that emotional price and emotional experience can positively predict consumers’ purchase intention, but the emotional product and purchase intention are negatively correlated, thus H1a and H1c are verified, and H1b is not supported. Then, emotional price and emotional experience can positively predict consumers’ trust, but cannot show the relationship between emotional product and trust, thus H2a and H2c are verified, and H2b is not supported.

We also found that perceived intrusion is negatively associated with trust. The experiment shows that there is no significant influence between perceptual monitoring and trust. There was no positive effect between information disclosure and trust. Thus H3b is verified, and H3a and H3c are not supported. Studies have shown that expectation confirmation has a positive correlation with consumers’ trust. However, it is not associated with consumers’ purchase intention. Thus H4 is verified, and H8 is not supported. Finally, trust is positively associated with consumers’ purchase intention, thus H5 is supported.

### 4.5. Mediation Test

We did the mediating test with Amos25, using 2500 resampling bootstrapping and the results are shown in Table 6. The indirect effects of emotional price on purchase intention via the trust are significant (0.313, p=0.008<0.01), and the direct effects of emotional price on purchase intention are not significant (0.211, p=0.416>0.01), which means that trust fully mediates the effect of emotional price on purchase intention. Thus, H6a is supported. The indirect effects of emotional products (0.071, p=0.449>0.01) and emotional experience (0.122, p=0.140>0.01) on purchase intention via the trust are not significant, which suggests they have no mediation effect. Thus, H6b and H6c are not supported. The indirect effects of expectation confirmation on purchase intention via trust are significant (0.403, p=0.014<0.05), and the direct effects of expectation confirmation on purchase intention are not significant (0.100, p=0.584>0.01), which means that trust fully mediates the effect of expectation confirmation on purchase intention. Thus, H8 is verified.

### 4.6. Calculation Results of the Model

**Figure 4:**
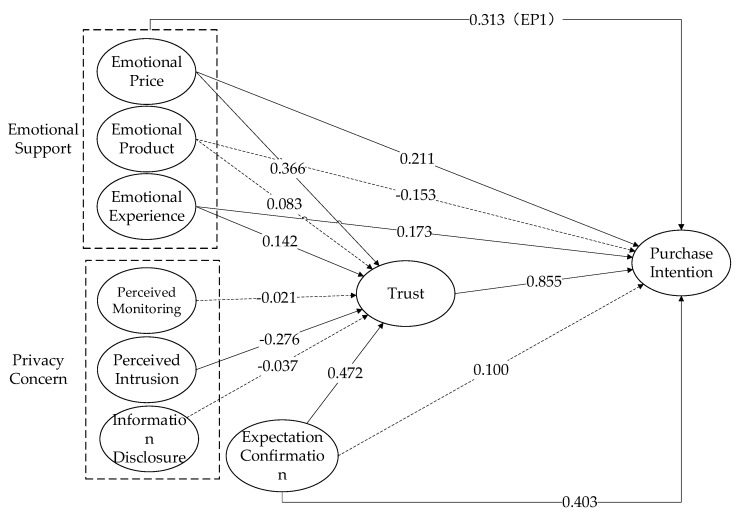
Model test diagram.

After calculation and arrangement, the model test diagram can be obtained (see the results in Figure 4).

## 5. Discussion

According to the hypothesis test of WeChat emotional support on trust, the following conclusions are drawn. Firstly, WeChat emotional price and emotional experience have a significant positive correlation with consumer trust. The hypotheses H1a, H1c, H2a, and H2c  are supported, which also confirm the previous studies which found the main reasons for users to buy medicines online are price and convenience [48,50,51]. These results suggest that a product price strategy might improve consumers’ trust in products or enterprises. The improvement of product price strategy based on the WeChat platform could encourage consumers to establish a trust in enterprise product information, so as to enhance consumers’ purchase intention. Consumers’ emotional experience could also affect their purchase intention. Therefore, enterprises should improve the timeliness and effectiveness of WeChat system communication. At the same time, enterprises might strengthen the information quality of WeChat health products, optimize the operation interface of WeChat, and give full play to the emotional support effect of WeChat in healthcare management.

In the study, WeChat emotional products are negatively associated with purchase intention and it has nothing to do with consumer trust. It might mean that consumers’ trust in products does not come from the exquisite packaging and design of products. Moreover, the better the packaging and design of products, the lower the purchase intention of consumers. This finding in the paper contradicts the results of previous research. Previous studies have shown that sensory experience of online products will affect purchasing behavior [37,38]. A previous study has also found that food packaging design will affect consumers’ preferences and purchase intention [97]. One possible explanation is that the packaging design of products would have different effects on consumers’ purchasing intentions due to the functions of different products. Moreover, the interviews and data of this research come from the purchasing experience of Chinese social network consumers. In recent years, health products including traditional Chinese medicine have also been sold on WeChat social network. Health products emphasize the special features of product safety and effectiveness. The packaging and design of products are not the focus of consumers buying health products online. Therefore, if enterprises invest too much in the packaging design of healthy products, it may cause the aversion of consumers. Consumers may think that companies should spend less on improving the health and treatment effectiveness of their products. Therefore, this may lead to a decline in consumers’ willingness to purchasing. This further suggests that Chinese consumers will be more willing to recognize and buy products from traditional Chinese medicine companies that are more than 100 years old, rather than emerging pharmaceutical companies. As a result, the new finding also indicates that the research result of this paper is a beneficial supplement to the research on the purchase intention behavior of network products.

The research finds that privacy invasion is negatively correlated with trust. It indicates that trust might reduce when users perceive the risk of invasion in the business system. After consumers register as system users, they hope that the information registered in the system can not release. This means that enterprises should strengthen security guarantees in health trading system to avoid personal information obtained by an unknown party. At the same time, the results of this paper show that perception monitoring and perception information disclosure will not affect the trust of users, which is contrary to the previous hypothesis. In China, the public might not be aware of the protection of personal privacy information and pays little attention to privacy. On the one hand, Chinese netizens might believe that private security comes more from the security guarantee provided by the registered business system. Therefore, the privacy invasion has a negative correlation with trust. On the other hand, in the use of mobile social services, people’s positive perception of the usefulness of products and systems is much higher than their negative perception of privacy concerns. Hence, consumers would like to risk their privacy for a better online shopping experience.

Expectation confirmation is the perception that a user’s expectations which is consistent with the reality of using social media services. If an individual expectation matches the actual performance of WeChat service, it would increase the trust of users and thus increase the purchase intention. In the study, the expectation confirmation has a significant positive effect on trust, and users with higher trust will have stronger purchase intention. The conclusion of this study might indicate that health services based on WeChat should also attach importance to the target of customer expectation, continuously improve user trust, and thus enhance user stickiness.

This paper verifies that trust fully mediates the relationship between emotional price and purchase intention. However, no evidence shows that trust plays a mediating role between emotional products and purchase intention. In the test, the mediating effect of trust between emotional experience and purchase intention is not verified. These results may be interpreted by the fact that the cognitive behavior of buying health products online is really different from other products. Because health products are very important to personal health and even life safety. So, buying health products on the WeChat platform, trust is particularly important for purchase intention. Meanwhile, trust plays a complete mediating role between expected confirmation and purchase intention. When consumers’ expectation of products is consistent with reality, consumers’ trust in products might be enhanced, and their purchase intention will also be enhanced. The findings confirm that consumers’ trust in health products is also a psychological expectation, such as the efficacy, safety, and authority of health products. Therefore, emotional marketing will enhance the trust of consumers and enhance corporate reputation and brand identity.

## 6. Conclusions

In China, mobile social media has been widely used in the field of health product consumption, which has had an impact on netizens’ healthy behaviors. Social media marketing of medical care products can play an active role in promoting public health. It can not only achieve precision marketing, improve the brand awareness of health products, but also play a positive role in personalized health care management. The social network platform represented by WeChat has a profound impact on public healthcare management. To sum up, this paper analyzes the status of China’s health industry in the new media economic context. With a view to the emotional and cognitive needs of WeChat consumers, the influence model of purchase intention of health products was constructed. This study analyzes how emotional support, expectation confirmation, privacy concern, and trust have an effect on consumers’ purchase intention in WeChat. It can be seen from this study that attaching importance to users’ emotional support, providing a safe private environment and improving expectations for healthcare management is the basis for the long-term development of social media healthcare management on social media.

Firstly, the academic significance of this study lies in: the “satisfaction” in the expectancy confirmation model is replaced by “trust” because consumers buy health products, trust can better express the recognition of the quality and brand of health products than satisfaction. Moreover, the test of intermediate variables in this paper also proves that emotional input and meeting consumers’ expectations for products will be conducive to the establishment of consumers’ trust and thus affect their purchase intention. This also shows that trust as a mediator variable is appropriate in this article. In addition, the influence relationship between expectation confirmation and trust in this paper is consistent with that of previous scholars [72,73].

Secondly, this paper analyzes the influence of emotional price, emotional products and emotional experience on trust and purchase intention. It is worth noting that the results of emotional products of this study are different from previous studies. We find that emotional products are negatively correlated with purchase intention and it has no impact on consumer trust. The reasons for this contradiction may be as follows: (1) The research object of this paper is online health products sold on social media. Due to different characteristics from other online products of the previous study, respondents in this study could pay more attention to the treatment and health care effect of health products. Therefore, the design and packaging of health products have different influences on consumers’ purchasing intention than other online products. (2) The purchase habits and social consumption culture of the respondents in the study are different. The respondents of this study are consumers of Chinese social network, who expect more effect of product treatment and health care than product packaging design.

Thirdly, this paper examines the sufficient mediating effect of trust in emotional price and purchase intention and the sufficient mediating effect of trust in expectation confirmation and purchase intention. The test of the intermediate variable also proves that enterprises’ emotional input and meeting consumers’ expectations for products are conducive to the establishment of consumer trust [79]. Therefore, the results of the study further verified the improved model in this paper that replacing the mediating variable with trust instead of satisfaction variable would be more in line with consumer’ cognitive needs of buying health products online. Previous studies have shown that emotional experience and packaging design of products have an increasing influence on trust and purchase [24]. However, this paper finds that trust does not mediate between emotional products, emotional experience, and purchase intention. So, the results also show that the cognitive behavior of buying online health products is indeed different from other online products. Consumers may pay special attention to the expected efficacy of healthy products, and their purchase intention may be more influenced by the price and expectation of products (to some extent, the price represents the research and development investment of healthy products). In contrast, it is difficult to build consumer trust in healthy products of packaging design and emotional experience.

The study further verifies the applicability of privacy concern scale in China’s mobile network. Another important finding is that Chines consumers seem not to pay that much attention to privacy concerns, only when they perceive that privacy information has been intruded will their trust in products be reduced. Previous studies have shown that privacy information disclosure will cause customer dissatisfaction and distrust [46,47]. We think there may be two reasons for this: (1) In China, netizens’ awareness of privacy protection is not strong, which leads to the fact that even if personal information is leaked, people will not care too much about it. (2) The existence of the privacy paradox, when people want to obtain certain benefits, they can allow their private information to be disclosed [98]. Therefore, in order to buy health products and obtain better service experience, people are willing to accept a certain degree of privacy disclosure. On the whole, our research reflects Chinese netizens’ attitudes towards privacy concerns.

Moreover, there are still other factors that could affect consumer behavior. Follow-up studies can further explore whether there are other mediating variables besides trust in WeChat healthy consumption, so as to have a more comprehensive understanding of healthy consumption behavior of consumers. In recent years, the online family health market in China has been developing rapidly. Meanwhile, the post-90s generation attaches more importance to their healthcare management [99]. Therefore, in the follow-up research, we try to take online family health consumers as the research object and use big data technology to accurately analyze the trend and group behavior characteristics of family health consumption. With the growth of the Internet, the post-90s generation has become the main force of online consumption, and they have a stronger sense of responsibility for themselves and their families. In the follow-up research, we can try to add responsibility as an intermediary variable to further fully understand the influence of online healthy consumers’ rational cognition on consumption behavior.

Despite these inherent limitations, the healthcare industry will be the most important increment in China in the future. The medical and health industry constructed by social media has gradually formed an important environment for health communication and healthy consumption. In order for the public to fully enjoy the benefits of the digital health industry, health service providers must give priority to issues related to trust. Trust is a key way to promote public acceptance of health services and purchase of health products on the Internet.

## Figures and Tables

**Table 1:** Summary of specific measurement questions of questionnaire variables.

Variable/Abbreviation		Descriptive	Adapted From:
Emotional Support (ES)	Emotion Price (EP1)	Q1(EP1)- The product price is in line with my psychological price in online shopping.	Lee (2017) [81] Brijnath (2015) [50]
		Q2(EP1)- I will pay attention to promotions and discounts information in online shopping.	
		Q3(EP1)- l can discuss the product price with the seller in online shopping.	
	Emotion Product (EP2)	Q1(EP2)- The packaging and design of online products have a strong sensory impact on customers.	Pentus (2014) [24] Lee (2018) [49]
		Q2(EP2)- The packaging and design of online products deliver the value of the brand.	
		Q3(EP2)- Packaging and design of online products make me feel healthy.	
	Emotion Experience (EE)	Q1(EE)-The navigation of the application in the WeChat interface is clear and easy to understand.	Park (2012) [51] Hwang (2010) [47]
		Q2(EE)-The function of the WeChat enables me to accomplish a shopping task more quickly than other ways of shopping.	
		Q3(EE)- Customer services on WeChat are friendly and can solve my doubts.	
Privacy Concern (PC)	Perceived Monitoring (PM)	Q1(PM)- This system could monitor my location through my purchasing behavior.	Dienlin (2015) [91] Henke (2018) [92]
		Q2(PM)- The system could collect too much personal information from transactions.	
		Q3(PM)- This system could monitor the usage of my mobile phone. through my purchasing behavior.	
	Perceived Intrusion (PI1)	Q1(PI1)- I am afraid that others get more my privacy through this system than they are allowed.	James (2017) [93] Demmers (2018) [29]
		Q2(PI1)- I would be concerned that the information transmitted through online transactions could be intercepted by third parties.	
		Q3(PI1)- I would be worried about the security of the system by hackers login access to my personal data.	
	Information Disclosure (ID)	Q1(ID)- I could be concerned that the system may use private information for other purposes without authorization.	Kim (2008) [17] Yoonhyuk (2018) [61] Hallikainen (2018) [78]
		Q2(ID)- I could be concerned about the system selling private information to others without permission.	
		Q3(ID)- I could be worried that the system will share my private information with others without my authorization.	
		Q1(EC)- My experience in using this system was better than what I had expected.	Bhattacherjee (2001) [94]
	Expectation Confirmation (EC)	Q2(EC)- The product and service provided by this system were better than what I had expected.	
		Q3(EC)- Overall, most of my expectations from using this system were confirmed.	
		Q1(T)- The interface design of the system is clear, professional and distinct, which will give customers a real feeling.	
	Trust (T)	Q2(T)- Sellers actively maintain communication with customers, which reflects the importance of customers.	Kim (2012) [73] Oghuma (2016) [95]
		Q3(T)- WeChat system can share the information of buyers’ feedback on products, which makes me feel credible.	
	Purchase Intention (PI)	Q1(PI)- I am likely to purchase the products on this business system.	Lankton (2014) [43] Fang (2014) [74]
		Q2(PI)- I am likely to recommend this bustiness system to my friends.	
		Q3(PI)-I am likely to make repurchase from this system.	

**Table 2:** Discriminant validity test.

Item	EP1	EP2	EE	PM	PI1	IL	EC	T	PI
EP1	**0.742**								
EP2	0.609	**0.801**							
EE	0.647	0.732	**0.777**						
PM	0.137	0.210	0.192	**0.767**					
PI1	0.019	0.090	0.141	0.568	**0.805**				
IL	0.165	0.219	0.243	0.621	0.735	**0.871**			
EC	0.616	0.632	0.584	0.024	−0.580	0.019	**0.843**		
T	0.705	0.626	0.584	−0.019	−0.019	−0.110	0.717	**0.794**	
PI	0.674	0.579	0.579	−0.027	−0.151	0.008	0.670	0.709	**0.769**

Note: Off-diagonal: correlation estimated between the factors. Diagonal (bold): square root of AVE. The abbreviations for variables are defined in Table 1.

**Table 3:** Reliability and validity test.

Variable	Cronbach α	Factor Loading	C.R.	AVE
EP1	0.781	0.699	0.7853	0.5504
		0.842		
		0.719		
EP2	0.835	0.880	0.8416	0.6423
		0.666		
		0.842		
EE	0.820	0.755	0.8208	0.6044
		0.790		
		0.787		
PM	0.804	0.672	0.8087	0.5876
		0.865		
		0.750		
PI1	0.801	0.871	0.8451	0.6483
		0.861		
		0.667		
ID	0.899	0.954	0.9034	0.7581
		0.797		
		0.854		
EC	0.829	0.807	0.8304	0.7104
		0.877		
		0.825		
T	0.889	0.732	0.8363	0.6307
		0.817		
		0.830		
PI	0.861	0.820	0.8124	0.5914
		0.761		
		0.723		

Note: AVE is the average variance extracted. C.R. is the composite reliability. The abbreviations for variables are defined in Table 1.

**Table 4:** Model fitting parameters.

Index	Model Value	Recommended Value	Acceptance
X^2^/df	3.548	<3 good fit	reasonable
		<5 reasonable fit	
RSMEA	0.091	<0.05 good fit,	reasonable
		<0.1 reasonable fit	
SRMR	0.086	<0.05 good fit,	reasonable
		<0.1 reasonable fit	
NFI	0.862	Close to 1	reasonable
CFI	0.928	Close to 1	good
IFI	0.831	Close to 1	reasonable
AIC	702.000	the smaller the better	reasonable
ECVI	7.852	the smaller the better	reasonable

Note: RMSEA is the root mean square error of approximation. SRMR is the standardized root mean square residual. NFI is the normed fit index. CFI is the comparative fit index. IFI is the incremental fit index. AIC is the Akaike information criterion. ECVI is the expected cross-validation index.

**Table 5:** Structural model relationships obtained.

Hypothesis	Estimate	C.R.	*p*-Value	Results
H1a:EP1 => PI	0.211	2.288	*	Supported
H1b:EP2 => PI	−0.153	−2.545	*	Not supported
H1c:EE => PI	0.173	2.448	*	Supported
H2a:EP1 => T	0.366	5.305	***	Supported
H2b:EP2 => T	0.083	1.619	0.106	Not supported
H2c:EE => T	0.142	2.407	*	Supported
H3a:PM => T	−0.021	−0.326	0.744	Not supported
H3b:PI1 => T	−0.276	−3.299	***	Supported
H3c:ID => T	−0.037	−0.645	0.519	Not supported
H4:EC => T	0.472	6.183	***	Supported
H5:T => PI	0.855	4.810	***	Supported
H7:EC => PI	0.100	0.946	0.344	Not supported

Note: * *p* < 0.05, ** *p* < 0.01, *** *p* < 0.001. C.R. is the composite reliability.

**Table 6:** Mediation test obtained.

Hypothesis	Indirect	Direct	Total	Mediation
H6a:EP1=> T => *p*	0.313 **	0.211 (NS)	0.525 ***	Supported (Fully mediation)
H6b:EP2=> T => *p*	0.071 (NS)	−0.153 (NS)	−0.082 (NS)	Not supported
H6c:EE=> T => *p*	0.122 (NS)	0.173 (NS)	0.195 (NS)	Not supported
H8:EC => T=> *p*	0.403 *	0.100 (NS)	0.504 **	Supported (Fully mediation)

Note: * *p* < 0.05, ** *p* < 0.01, *** *p* < 0.001.
